# Sustainable orthodontics: direct environmental, material, and implementation evidence from a systematic review

**DOI:** 10.3389/froh.2026.1772579

**Published:** 2026-10-14

**Authors:** Mohammad Khursheed Alam, Suliman Alsaeed, Mohammad Younis Hajeer, Huda Abutayyem, Afnan Alayyash, Khulood Alhajeri

**Affiliations:** 1 Preventive Dentistry Department, College of Dentistry, Jouf University, Sakaka, Saudi Arabia; 2 Department of Dental Research Cell, Saveetha Institute of Medical and Technical Sciences, Saveetha Dental College and Hospitals, Chennai, India; 3 Department of Public Health, Faculty of Allied Health Sciences, Daffodil International University, Dhaka, Bangladesh; 4 Preventive Dental Sciences Department, College of Dentistry, King Saud bin Abdulaziz University for Health Sciences, Riyadh, Saudi Arabia; 5 King Abdullah International Medical Research Center, Riyadh, Saudi Arabia; 6 Ministry of the National Guard—Health Affairs, Riyadh, Saudi Arabia; 7 Department of Orthodontics, Faculty of Dentistry, University of Damascus, Damascus, Syria; 8 Department of Clinical Sciences, Center of Medical and Bio-Allied Health Sciences Research, College of Dentistry, Ajman University, Ajman, United Arab Emirates; 9 Preventive Dentistry Department, Taif University, Taif, Saudi Arabia; 10 Emirates Health Services, Ras Alkhaimah Specialist Center, Nokhbat Alemarat Medical Center, Ras Al Khaimah, United Arab Emirates

**Keywords:** clear aligners, environmental sustainability, implementation, life-cycle assessment, orthodontics, retainers, systematic review, waste disposal

## Abstract

Orthodontic care generates environmental burdens through polymer and metal consumption, single-use products, manufacturing, transport, energy use, and end-of-life disposal. This systematic review evaluated primary orthodontic evidence that directly measured environmental outcomes or examined implementation behavior explicitly related to environmental sustainability. PubMed/MEDLINE, Scopus, Web of Science Core Collection, EMBASE, and the Cochrane Library were searched from January 2010 through October 2025, with supplementary Google Scholar and reference-list screening. The corrected search used two reproducible concept blocks—orthodontics and environmental sustainability—without a human-subject filter so that laboratory and life-cycle studies remained eligible. Five primary studies met the revised PICOS criteria: two laboratory/material studies, one comparative life-cycle assessment, and two cross-sectional surveys. Incineration of used clear-aligner polymers produced measurable hazardous emissions; a comparative life-cycle assessment indicated that retainer selection was sensitive to product lifespan and system-boundary assumptions; and a shape-memory aligner material showed potential to reduce the number of appliances, although a complete life-cycle assessment was not performed. Survey evidence showed inadequate disposal knowledge and a persistent gap between favorable attitudes and routine sustainable practice. The evidence remains sparse, heterogeneous, and at some risk of selection, modeling, and self-report bias. Current findings support targeted action on aligner and retainer life cycles, disposal guidance, and implementation barriers, but stronger orthodontic-specific life-cycle studies and standardized environmental outcomes are required. Because eligibility was intentionally restricted to primary orthodontic studies with direct environmental outcomes or explicit sustainability-related implementation outcomes, the five included studies represent a highly selected evidence base rather than a comprehensive survey of all sustainability-related orthodontic literature. The 4D-printing finding is interpreted only as a potential appliance/material-reduction pathway; no net environmental benefit has yet been demonstrated.

**Systematic Review Registration**: https://www.crd.york.ac.uk/PROSPERO/view/CRD420251250285, identifier CRD420251250285.

## Introduction and background

Orthodontic care has traditionally been evaluated according to effectiveness, safety, patient experience, and technological performance. Its environmental consequences are less consistently measured, even though treatment requires metals and polymers, single-use barriers, sterilization, electricity, transport, packaging, and repeated clinical visits. Healthcare contributes a material share of global greenhouse-gas emissions, and dental supply chains, travel, energy consumption, and waste are recognized contributors (1, 2). Sustainable orthodontics therefore requires evidence that distinguishes direct environmental benefit from assumptions based only on clinical novelty or material performance.

A life-cycle perspective considers raw-material extraction, manufacturing, clinical use, transport, replacement frequency, and end-of-life management. Digital and additive workflows may reduce some physical impressions and model waste, but they can also introduce resin consumption, electricity demand, device turnover, data storage, and electronic waste (3, 4). Earlier orthodontic sustainability literature proposed the four-R framework—rethink, reduce, reuse, and recycle—while emphasizing that environmental gains must not compromise infection control or treatment quality (5).

Recent narrative publications, including the World Federation of Orthodontists white paper and broader dental sustainability reviews, have mapped likely environmental pressures and implementation barriers (6–8). However, narrative discussions frequently combine direct environmental measurements with indirect clinical, microbiological, or material outcomes. The present review was therefore refocused to include only primary orthodontic studies that measured a direct environmental outcome, evaluated an intervention explicitly intended to reduce environmental burden, or investigated orthodontic sustainability behavior and implementation. This narrower scope restores a clear scientific contribution and avoids treating clinical benefit alone as evidence of sustainability.

## Review

### Methodology

This systematic review was conducted and reported in accordance with the Preferred Reporting Items for Systematic Reviews and Meta-Analyses (PRISMA) 2020 statement (9). The protocol was registered in PROSPERO (CRD420251250285). The protocol allowed quantitative pooling when sufficiently comparable outcomes were available; because the eligible studies differed in design, functional unit, intervention, and outcome metric, the final synthesis was narrative.

#### Rationale and execution of the systematic literature search

The search strategy was revised for reproducibility and consistency with the PICOS criteria. It used two core concept blocks: (1) orthodontics, appliances, aligners, retainers, and orthodontic workflows; and (2) environmental sustainability, life-cycle assessment, carbon footprint, waste, disposal, recycling, biodegradability, resource use, and circular economy. The previous third block based on generic dental materials and practice terms was removed because it reduced sensitivity and could exclude relevant life-cycle and disposal studies. No human-subject filter and no publication-type filter were applied during database searching, because eligible evidence included laboratory environmental testing, material studies, life-cycle assessments, and surveys. Exact field tags and database-specific syntax are presented in Table 1. The final syntax used controlled vocabulary where available (for example, MeSH in PubMed and Emtree in EMBASE) together with broad free-text roots and phrases such as sustainab*, “carbon footprint,” “life cycle assessment,” recycl*, waste, disposal, biodegrad*, and “circular economy.” Emerging labels such as green dentistry, sustainable materials, and digital orthodontics were not imposed as separate mandatory concepts because the final strategy prioritized sensitivity to direct environmental outcomes. No formal PRESS-style independent peer review of the search strategy is reported; the complete database-specific strings are therefore provided so that readers can directly appraise and reproduce the search.

**Table 1:** Search strategy and syntax for identifying studies on sustainable orthodontics.

Database/source	Reproducible search syntax	Limits	Fields/notes
PubMed/MEDLINE	((“Orthodontics”[Mesh] OR orthodontic*[tiab] OR “clear aligner*”[tiab] OR retainer*[tiab] OR brace*[tiab]) AND (sustainab*[tiab] OR “environmental impact”[tiab] OR “carbon footprint”[tiab] OR “life cycle assessment”[tiab] OR lifecycle[tiab] OR recycl*[tiab] OR waste[tiab] OR disposal[tiab] OR biodegrad*[tiab] OR “circular economy”[tiab]))	2010–Oct 2025; English	No human or publication-type filter
Scopus/Web of Science	TITLE-ABS-KEY/TS = ((orthodontic* OR “clear aligner*” OR retainer* OR brace*) AND (sustainab* OR “environmental impact” OR “carbon footprint” OR “life cycle assessment” OR lifecycle OR recycl* OR waste OR disposal OR biodegrad* OR “circular economy”))	2010–Oct 2025; English	Title, abstract, keywords/topic
EMBASE	(orthodontics/exp OR orthodontic*:ti,ab,kw OR “clear aligner*”:ti,ab,kw OR retainer*:ti,ab,kw) AND (environmental sustainability/exp OR sustainab*:ti,ab,kw OR “life cycle assessment”:ti,ab,kw OR “carbon footprint”:ti,ab,kw OR recycl*:ti,ab,kw OR waste:ti,ab,kw OR disposal:ti,ab,kw)	2010–Oct 2025; English	Emtree plus title/abstract/keyword
Cochrane Library	([mh Orthodontics] OR orthodontic*:ti,ab,kw OR “clear aligner*”:ti,ab,kw OR retainer*:ti,ab,kw) AND (sustainab*:ti,ab,kw OR “environmental impact”:ti,ab,kw OR “life cycle assessment”:ti,ab,kw OR “carbon footprint”:ti,ab,kw OR waste:ti,ab,kw OR disposal:ti,ab,kw)	2010–Oct 2025; English	Trials and reviews screened; only eligible primary studies retained
Google Scholar and hand search	"sustainable orthodontics” OR “orthodontic environmental impact” OR “clear aligner disposal” OR “orthodontic life cycle assessment” OR “orthodontic carbon footprint”	First 200 results	Sorted by relevance; reference lists and key reviews checked

The electronic searches covered January 2010 through October 2025. Google Scholar screening was limited prospectively to the first 200 results sorted by relevance, and reference lists of included studies and key orthodontic sustainability reviews were checked. Two reviewers independently screened titles/abstracts and then full texts. Disagreements were resolved by discussion and, when necessary, adjudication by a third reviewer. Search counts in the PRISMA diagram correspond to the documented search run and may vary slightly when the same syntax is rerun after database indexing updates.

### Application of the PICOS framework for study selection

Eligibility was defined using a revised PICOS framework (10) (Table 2). Comparator requirements were made design-appropriate: a conventional material, alternative pathway, or baseline was required for comparative studies, whereas descriptive emission studies and cross-sectional implementation surveys were permitted without an artificial comparator. Direct environmental outcomes were prioritized; clinical or mechanical outcomes were eligible only when they formed part of an intervention explicitly designed to reduce environmental burden. These deliberately narrow criteria were intended to maximize conceptual specificity for direct environmental evidence. They consequently exclude a broader literature on clinical innovation, biomaterials, digital orthodontics, and general green-dentistry concepts when environmental outcomes were not directly measured. The resulting evidence base should therefore be interpreted as highly selected rather than as a comprehensive representation of all sustainability-related orthodontic research.

**Table 2:** Revised study eligibility criteria (PICOS framework) (10).

PICOS element	Inclusion criteria	Exclusion criteria
Population/setting (P)	Orthodontic patients, orthodontists, orthodontic teams, orthodontic appliances/materials, retention, or orthodontic clinical/manufacturing workflows.	General dentistry without extractable orthodontic data; animal studies without an orthodontic environmental question.
Intervention/exposure (I/E)	An intervention, material, workflow, disposal route, or professional behavior explicitly intended to reduce environmental burden, or a study directly measuring the environmental impact of orthodontic care.	Clinical or material innovations with no stated environmental objective and no direct environmental outcome.
Comparator (C)	Conventional or alternative material/workflow, baseline, different disposal pathway, or modeled scenario when applicable. Comparator not mandatory for descriptive emission studies or cross-sectional implementation surveys.	Comparative claims without a defined alternative, baseline, or functional unit when the design requires one.
Outcomes (O)	Primary: carbon/greenhouse-gas emissions, energy, water, resource use, waste, toxic emissions, recyclability, biodegradability, life-cycle impacts. Secondary: cost, feasibility, knowledge, behavior, and clinical/material performance when explicitly linked to sustainability.	Only microbiological, antimicrobial, salivary, toxicological, bond-strength, or clinical outcomes without a direct environmental link.
Study design (S)	Primary empirical LCA, laboratory environmental testing, sustainability-oriented material studies, comparative observational studies, cross-sectional surveys, and mixed-method studies.	Reviews, editorials, opinions, letters without original data, protocols, conference abstracts without sufficient data, and non-peer-reviewed webpages.

#### Structured data extraction process

Two reviewers independently extracted author, year, country, design, orthodontic setting or material, functional unit or sample, intervention/exposure, comparator where applicable, direct environmental outcome, implementation outcome, and the principal numerical findings. For life-cycle assessments, extraction also included system boundaries, data sources, impact categories, product lifespan assumptions, and sensitivity analyses. Disagreements were reconciled by consensus.

#### Critical appraisal of evidence and bias assessment

Methodological appraisal was matched to study design rather than applying one observational tool to all studies. Dental *in vitro* studies were assessed using the QUIN tool (11); analytical cross-sectional surveys were assessed using the JBI checklist (12); and life-cycle assessments were evaluated against core ISO 14040 principles, including goal and scope, functional unit, system boundaries, inventory transparency, impact assessment, interpretation, limitations, and sensitivity analysis (13). Judgments were mapped to common domains for graphical presentation as low risk/adequate or some concerns. No study was excluded solely on appraisal score. For cross-design presentation, judgments were harmonized only across conceptually comparable domains: clearly stated aim; sample or functional-unit selection; intervention/exposure definition; outcome measurement; analysis/confounding; reporting transparency; and overall appraisal. “Low risk/adequate” indicates that the relevant criteria for that common domain were substantially met, whereas “some concerns” indicates one or more limitations. This mapping was descriptive and was not intended to convert QUIN, JBI, and ISO 14040 into a common numerical score.

Because the five studies differed markedly in design and outcome and included laboratory, life-cycle assessment, and cross-sectional evidence, a formal GRADE-type certainty assessment was not applied. Overall confidence in broad, practice-wide sustainability conclusions is limited: individual studies can support their directly measured findings within their own settings, but the sparse evidence base, design-specific limitations, and lack of replication reduce certainty and generalizability across orthodontic settings.

#### Narrative synthesis plan

Findings were synthesized using SWiM and narrative-synthesis guidance (14, 15). Studies were grouped into: (1) direct environmental burden and end-of-life evidence; (2) sustainability-oriented material innovation; and (3) practitioner knowledge, behavior, and implementation. Direction of findings, numerical results, methodological limitations, and applicability were compared within each domain. Meta-analysis, statistical heterogeneity estimates, and publication-bias tests were not performed because the outcomes and study designs were not commensurable.

## Results

### Study selection process

The documented search identified 680 records. After removal of 245 duplicates, 80 records marked ineligible by automation tools, and 15 records removed for other reasons, 340 records underwent title and abstract screening. Fifty-five full texts were sought, 10 could not be retrieved, and 45 were assessed for eligibility. Forty reports were excluded because they were reviews or commentaries, lacked a direct sustainability outcome, were not orthodontic in scope, or used an ineligible design. Five primary studies met the revised PICOS criteria and were included in the narrative synthesis (16–20) (Figure 1). The marked reduction from 680 records to five included studies reflects the deliberate exclusion of reviews/commentaries, non-orthodontic studies, and studies that discussed sustainability without directly measuring an environmental outcome or explicit sustainability-related implementation behavior (Table 3). Accordingly, the included set is intentionally specific rather than broadly representative.

**Figure 1:**
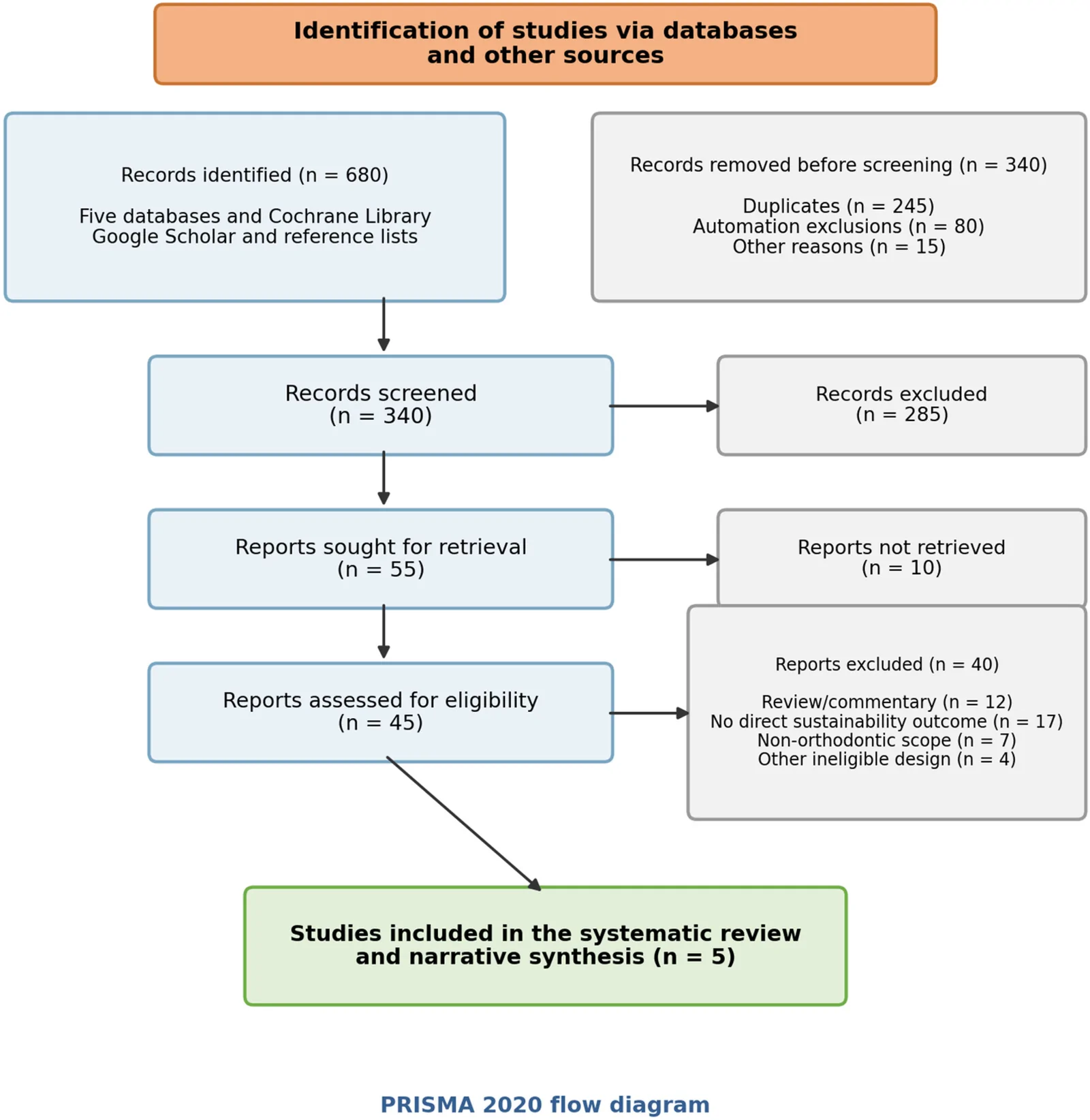
Revised PRISMA 2020 flow diagram of study selection.

**Table 3:** Representative full-text exclusions and reasons for exclusion.

Author(s), year	Reason for exclusion
Macri et al. (2024) (21)	Review article. Provides a useful narrative overview of aligner sustainability but does not report primary empirical data.
Ahmed et al. (2023) (22)	Narrative professional guidance. Discusses challenges and opportunities but does not present a primary study.
Peter et al. (2022) (23)	Special article/commentary. Raises concern about clear-aligner waste but does not report original environmental measurements.
Jasso-Ruiz et al. (2020) (24)	Outcome mismatch. Antimicrobial performance of silver-coated brackets was measured without a direct environmental outcome or sustainability analysis.
Dobrzanski et al. (2020) (25)	Scope/design mismatch. Broad conceptual discussion of sustainable dentistry rather than orthodontic primary evidence.
Hegedus et al. (2022) (4)	Population/scope mismatch for inclusion. General dental 3D-printing survey without orthodontic-specific results; retained only as background evidence.

Table 4 summarizes the five primary studies retained after aligning the search and eligibility criteria. The evidence comprised one clear-aligner incineration study, one comparative life-cycle assessment of post-orthodontic retainers, one *in vitro* study of a shape-memory aligner material intended to reduce appliance consumption, and two orthodontic sustainability surveys. The studies therefore address direct emissions, comparative product life cycles, material-reduction potential, disposal behavior, and implementation barriers. Geographic coverage was limited to a small number of national settings, which restricts transferability to healthcare systems with different energy sources, transport patterns, procurement systems, regulation, recycling access, and waste-management infrastructure.

**Table 4:** Characteristics and methodological overview of included studies.

Authors, year, country	Design	Material/participants	Sustainability exposure	Comparator	Principal findings
Peter et al. (2025), India (16)	Laboratory GC-MS study	Used PET-G and polyurethane clear aligners	Incineration/end-of-life emissions	Material types and combustion profiles	Hazardous volatile compounds were detected; incineration was not environmentally benign.
Da Tan et al. (2024), Ireland/UK (17)	Comparative LCA	Hawley and Essix retainers	Manufacture, in-house production, lifespan and replacement	Hawley vs. Essix retention	Hawley had higher production burden in 12/16 categories, but became more favorable when lifespan/replacement were modeled.
Palmieri et al. (2024), Italy (18)	*In vitro* material study	Shape-memory ClearX aligner specimens	4D-printing concept intended to reduce appliance number	Untreated vs. thermally activated/cycled specimens	Shape recovery was reversible and material performance was retained; net life-cycle benefit was not quantified.
Raj et al. (2024), India (19)	Cross-sectional survey	760 orthodontists/general dentists	Clear-aligner disposal knowledge and practice	Orthodontists vs. general dentists	Knowledge gaps and unsafe disposal persisted; orthodontists reported safer disposal more often.
Brierley et al. (2025), UK (20)	Cross-sectional survey	146 BOS members (10% response)	Knowledge, attitudes, practice, barriers and facilitators	Descriptive subgroup comparisons	High concern and willingness to change, but cost, regulation, policy and feasibility limited action.

GC-MS, gas chromatography-mass spectrometry; LCA, life-cycle assessment; PET-G, glycol-modified polyethylene terephthalate; BOS, British orthodontic society; CO2-eq: carbon dioxide equivalent.

The five included studies were organized into three domains: direct environmental burden and end-of-life management, sustainability-oriented material innovation, and practitioner knowledge and implementation. The revised synthesis deliberately excludes studies reporting only antimicrobial, microbiological, salivary, metal-release, or general dental outcomes because these do not directly establish environmental sustainability in orthodontics.

Peter et al. (16) directly examined the end-of-life consequences of clear aligners using GC-MS analysis of smoke generated by incinerating common thermoplastic materials. The study identified measurable hazardous volatile compounds from PET-G and polyurethane combustion. Its main contribution is direct evidence that routine incineration cannot be assumed to be environmentally benign; however, the experiment assessed selected materials and disposal conditions and did not compare a complete set of recycling or recovery pathways.

Da Tan et al. (17) conducted a comparative LCA of Hawley and Essix retainers across 16 impact categories. Hawley retainers had a higher production burden in 12 categories, but the conclusion changed after product lifespan and replacement frequency were incorporated, with Hawley retention modeled as having a lower environmental burden over the specified period under the stated lifespan and replacement assumptions. The study demonstrates why functional unit, durability, replacement, and system boundaries are central to orthodontic sustainability decisions. This comparison should not be generalized beyond the modeled context because LCA conclusions can change with geographical electricity mix, transportation distances and modes, inventory databases, waste-treatment pathways, functional unit, system boundaries, product lifespan, and replacement patterns.

Palmieri et al. (18) evaluated a shape-memory, 4D-printed ClearX aligner material through morphological, optical, thermomechanical, and cyclic testing. Heat-triggered shape recovery was reported to be reversible without loss of usability, supporting the concept that one appliance might deliver more than one programmed stage and thereby reduce material consumption. Nevertheless, the study did not perform a cradle-to-grave LCA; the environmental benefit therefore remains a plausible material-reduction pathway rather than a quantified net carbon or toxicity advantage. Accordingly, this finding is treated as evidence of material-function potential only; it does not demonstrate a reduced carbon footprint or a net environmental benefit.

Raj et al. (19) surveyed 760 orthodontists and general dentists about clear-aligner use and disposal. Orthodontists reported safer disposal practices more often than general dentists, but both groups demonstrated gaps in knowledge of biodegradation and disposal regulation. Regular garbage disposal and transfer of responsibility to patients remained common, showing that product design and recycling initiatives must be accompanied by clear manufacturer and professional guidance.

Brierley et al. (20) obtained responses from 146 British Orthodontic Society members (10% response rate). Environmental sustainability was widely regarded as important, and reduction of plastics, recycling of consumables, reduction of single-use items, and rethinking waste management were prioritized. Cost, regulation, organizational policy, and feasibility were prominent barriers. The low response rate and self-selection of environmentally interested respondents limit generalizability, but the study provides orthodontic-specific implementation evidence. These survey outcomes represent self-reported knowledge, attitudes, and practices rather than objectively measured environmental behavior. Membership/convenience sampling, non-response, and social-desirability bias may influence the estimates, and associations between reported barriers and practices should not be interpreted as causal.

Across domains, the evidence indicates a consistent problem definition but not a single intervention effect. Clear-aligner and retainer studies identify material, lifespan, and end-of-life hotspots; the 4D-printing study proposes a reduction pathway that still requires full LCA; and the surveys identify knowledge, policy, cost, and disposal infrastructure as reported factors associated with adoption of technical solutions. These outcomes are conceptually related but statistically non-combinable.

### Design-specific critical appraisal

Figure 2 summarizes the mapped appraisal domains. Aims and primary outcome measurement were generally clear, whereas concerns were concentrated in sample or functional-unit selection, modeling assumptions, confounding, response bias, and limited sensitivity analysis. All five studies were judged to have some concerns overall, but none was considered unusable for a cautious narrative synthesis.

**Figure 2:**
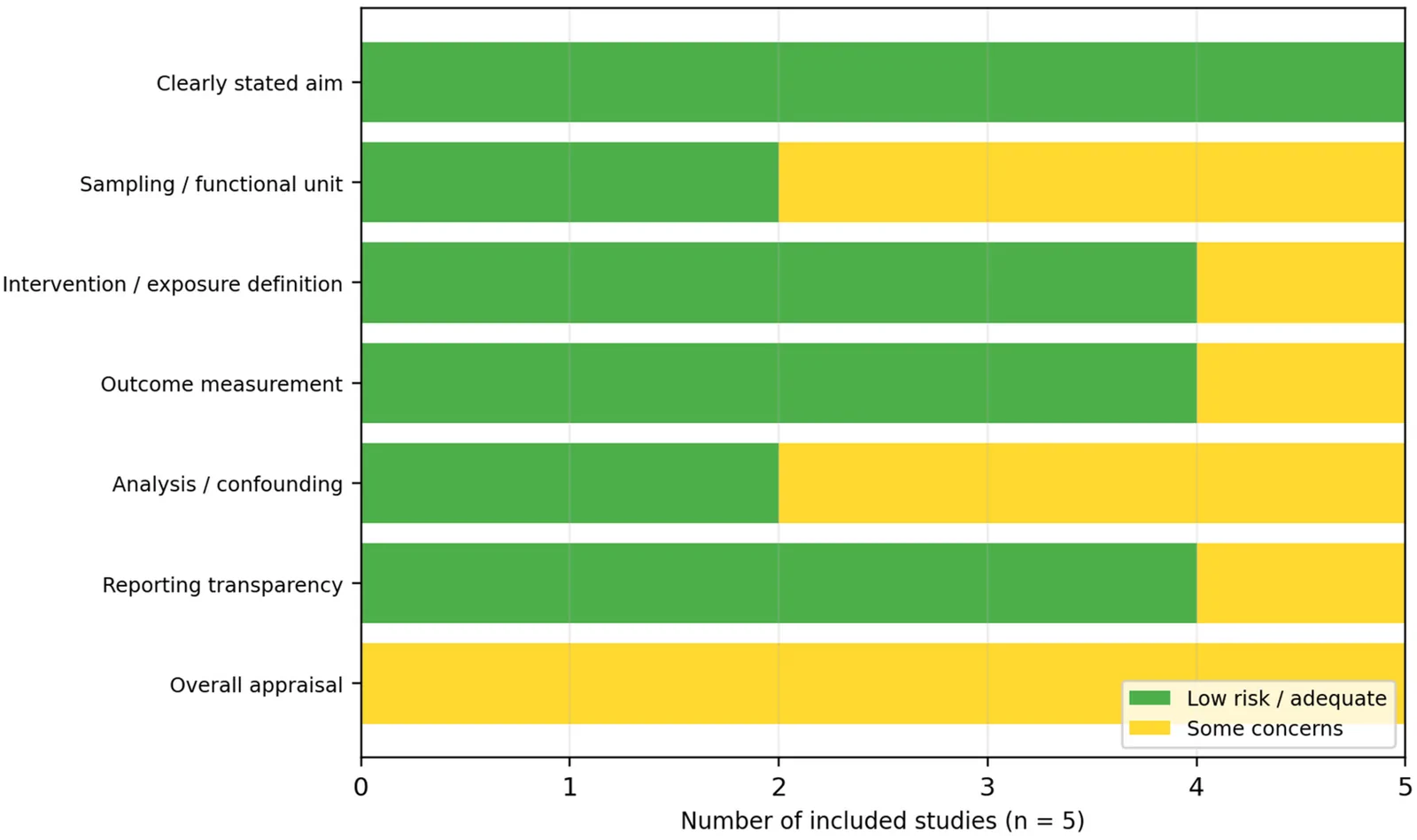
Summary of design-specific critical-appraisal judgments across the five included studies.

Figure 3 presents study-level judgments. The laboratory studies were limited mainly by material selection and the absence of complete life-cycle comparison. The LCA was transparent but sensitive to assumptions about lifespan, replacement, and inventory data. The surveys were limited by self-report, convenience or membership-based sampling, and non-response. These limitations restrict causal and policy conclusions while still permitting identification of environmental hotspots and implementation priorities.

**Figure 3:**
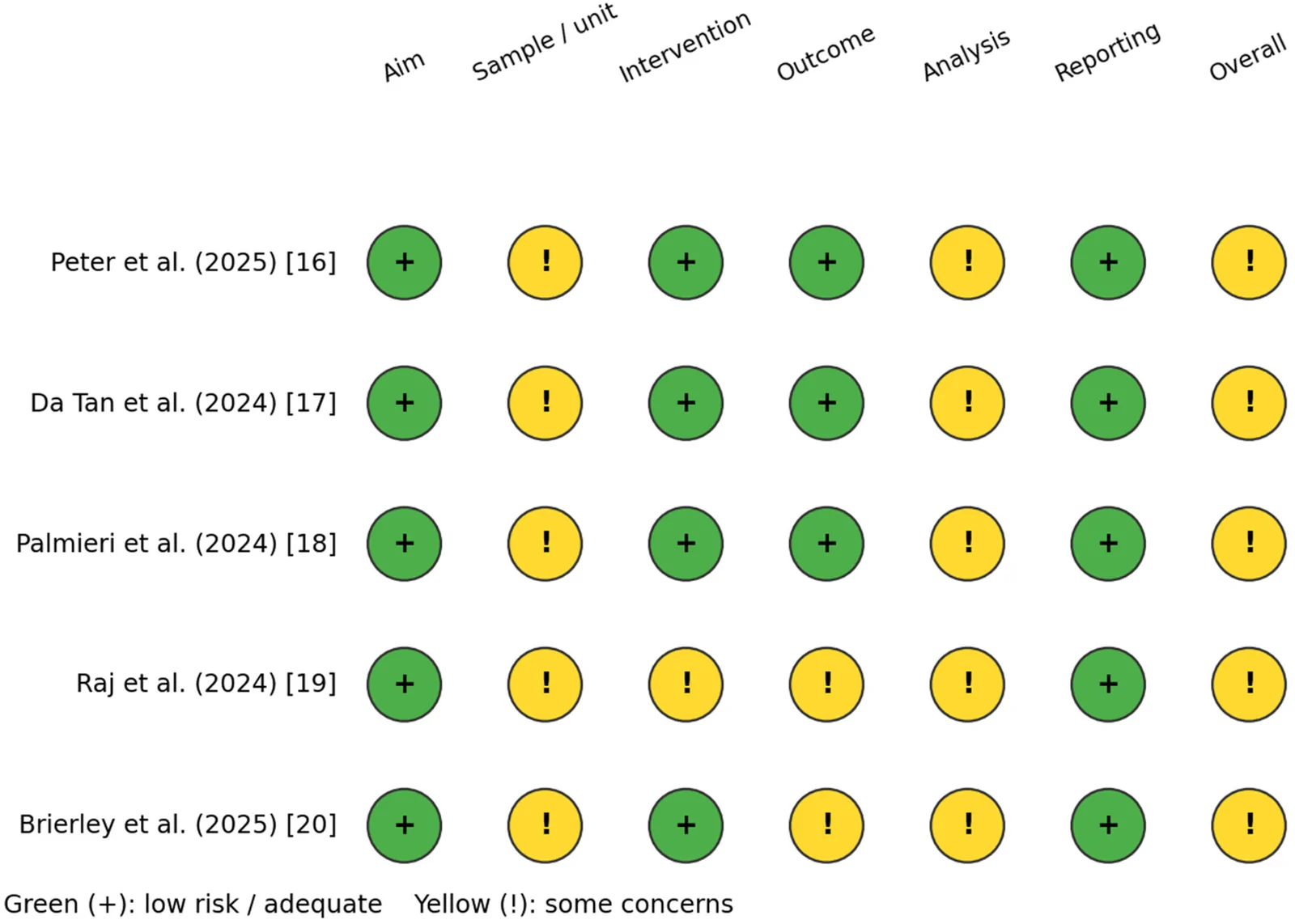
Study-level critical-appraisal matrix mapped from QUIN, JBI, and ISO 14040 domains.

## Discussion

This systematic review provides a focused synthesis of direct environmental, sustainability-oriented material, and implementation evidence in orthodontics. Restricting eligibility to studies with a direct environmental link substantially reduced the number of included studies, but it improved conceptual validity and prevented clinical or microbiological outcomes from being mislabeled as evidence of ecological sustainability. Accordingly, the review should be interpreted as a synthesis of highly selected direct or explicitly sustainability-oriented evidence, not as a comprehensive map of every orthodontic publication that mentions sustainability.

The findings complement the World Federation of Orthodontists white paper (6) and earlier orthodontic sustainability guidance (5, 22). Those publications describe the broad environmental responsibilities of the specialty; the present review adds a transparent distinction between narrative recommendations and primary empirical evidence. The small evidence base itself is an important finding: despite extensive discussion of sustainable orthodontics, only a few studies have directly measured emissions, life-cycle impacts, material-reduction potential, disposal behavior, or orthodontic implementation.

The strongest direct environmental evidence concerned orthodontic polymers and retention. Incineration testing showed that used aligner materials can generate hazardous emissions (16), while the comparative retainer LCA showed that a product with a larger manufacturing burden may still be preferable when longevity and replacement are incorporated (17). These studies caution against simple labels such as recyclable, digital, or plastic-free without specifying functional unit, system boundary, and end-of-life scenario.

Interpretation of LCA evidence requires particular caution because results are conditional on modeling choices. Geographic electricity generation, transportation distance and mode, manufacturing location, inventory-database selection, end-of-life assumptions, functional unit, system boundaries, and expected replacement frequency can materially alter comparative estimates. Therefore, even apparently similar appliance comparisons performed in different settings may not be directly comparable, and an environmentally preferable result in one modeled scenario should not be treated as a universal property of the appliance.

The 4D-printed aligner study (18) represents a clinically relevant innovation because extending appliance function could reduce the number of aligners and associated packaging. However, fewer appliances do not automatically guarantee a lower total footprint. Printing energy, resin synthesis, activation steps, failed prints, transport, device life, and disposal must be quantified. Sustainability claims for novel orthodontic materials should therefore combine mechanical and clinical testing with complete life-cycle assessment.

The two surveys provide complementary implementation evidence (19, 20). Disposal knowledge was incomplete, and favorable attitudes did not consistently translate into routine practice. Respondents and the survey context identified missing manufacturer instructions, limited recycling routes, infection-control requirements, cost, and organizational policy as perceived barriers. Education alone is unlikely to be sufficient unless procurement standards, waste pathways, and professional guidance make the sustainable option feasible. Because these studies are cross-sectional and self-reported, the identified barriers should be interpreted as perceived constraints or correlates of reported practice rather than as demonstrated causes of environmental outcomes.

Geographical generalizability is also limited. The included evidence originated from only a few countries and healthcare systems; although studies from India contributed to the evidence base, the review does not adequately characterize the diversity of lower-resource settings or the range of waste-management and regulatory contexts worldwide. Local energy generation, transport logistics, procurement structures, recycling access, infection-control requirements, and waste regulation may materially alter both LCA estimates and the feasibility of implementation.

The revised evidence base does not support broad claims that digital orthodontics, clear aligners, or a particular appliance category is universally sustainable. The available studies instead support intervention-specific decisions: avoid uncontrolled aligner incineration, incorporate lifespan and replacement into retainer selection, test material-saving technologies with full LCA, and improve disposal guidance and institutional support.

Meta-analysis was inappropriate because the studies measured fundamentally different outcomes: chemical emissions, multiple LCA impact categories, thermomechanical material behavior, disposal knowledge, and implementation attitudes. A pooled effect estimate would have no coherent interpretation. Narrative synthesis therefore preserves the clinical and environmental meaning of each evidence domain.

### Practical implications for clinicians and policymakers

For clinicians, the current evidence supports cautious, intervention-specific actions: clear-aligner incineration should not be assumed to be environmentally benign; retainer longevity and replacement should be considered alongside production burden; and material-saving technologies should remain described as promising until full life-cycle evidence is available. For institutions and policymakers, the survey findings support clearer disposal guidance and greater attention to procurement, recycling pathways, and manufacturer instructions. Take-back schemes, standardized procurement criteria, and other system-level measures remain proposals for future evaluation rather than established evidence-based interventions.

### Limitations of the study

This review has several limitations. Only five primary studies met the revised criteria, and the evidence included laboratory, modeling, and cross-sectional designs rather than prospective clinical environmental evaluations. The search was restricted to English-language reports and may have missed non-indexed technical or industry data. LCA conclusions depend on functional units, inventory databases, geographic energy mixes, assumed lifespan, and replacement patterns. The material study did not quantify net life-cycle benefit, and both surveys were vulnerable to self-selection and self-report bias. Because few comparable studies were available, publication bias and certainty of evidence could not be estimated statistically. The evidence is also geographically concentrated, and heterogeneity in study populations, materials, functional units, environmental endpoints, data sources, and methodological approaches limits direct comparison and transferability of individual findings. Restriction to English-language publications may have introduced language and geographic bias by missing locally published evidence, particularly from settings in which energy mixes, regulation, recycling infrastructure, and waste-management practices differ from those represented here. Formal certainty grading was not performed because the included evidence spans fundamentally different study designs and non-comparable outcomes; overall confidence in broad generalization is therefore limited.

### Future directions

Future orthodontic research should use prospective comparative LCAs with explicit functional units, system boundaries, local energy and transport data, uncertainty analysis, and clinically realistic replacement rates. Clear aligners, fixed appliances, retainers, packaging, remote monitoring, digital models, and reusable alternatives should be compared across manufacturing, clinical use, travel, and end-of-life stages. Material innovations should report both clinical performance and environmental inventory data. Implementation studies should test whether manufacturer instructions, take-back schemes, procurement standards, staff training, and policy changes measurably reduce waste or emissions rather than only improving attitudes.

## Conclusions

The direct evidence for sustainable orthodontics is limited but clinically relevant. Current studies identify clear-aligner disposal, retainer lifespan, appliance consumption, and implementation barriers as priority areas. Firm recommendations require standardized environmental outcomes, orthodontic-specific life-cycle assessment, and prospective evaluation of whether proposed innovations produce net environmental benefit without compromising care. These conclusions apply to the selected direct or explicitly sustainability-oriented evidence and should not be generalized to all orthodontic technologies, materials, or healthcare settings.

## Data Availability

The original contributions presented in the study are included in the article/Supplementary Material, further inquiries can be directed to the corresponding authors.
